# From Complex Shaping to Mirror Finish: Additive Manufacturing of Aerospace‐grade C_f_/SiC Space Optics

**DOI:** 10.1002/advs.202517980

**Published:** 2025-11-08

**Authors:** Buhao Zhang, Li Wang, Xiao Chen, Yuquan Wei, Huisheng Tian, Zhengren Huang, Xuejian Liu, Zhongming Chen, Jie Yin

**Affiliations:** ^1^ State Key Laboratory of High Performance Ceramics Shanghai Institute of Ceramics Chinese Academy of Sciences Shanghai 200050 China; ^2^ Faculty of Engineering University of Nottingham University Park Nottingham NG7 2RD; ^3^ College of Materials Science and Opto‐Electronic Technology University of Chinese Academy of Sciences Beijing 100049 China; ^4^ School of Physical Science and Technology ShanghaiTech University Shanghai 201210 China

**Keywords:** 3D printing, C_f_/SiC composites, mechanical properties, physical vapor deposition, thermal properties

## Abstract

Among additive manufacturing strategies, selective laser sintering (SLS) enables complex, resource‐efficient architectures, yet its application to aerospace‐grade ceramic composites is hindered by high sintering activation energies and fragile interfacial bonding. Herein, a hybrid route is established that couples SLS preform fabrication with interface‐engineered densification and thin‐film finishing, using carbon‐fiber reinforced silicon carbide (C_f_/SiC) as a model for lightweight space mirrors. To overcome the inherent limitations of conventional liquid silicon infiltration (LSI), a deliberately engineered pyrolytic carbon (PyC) interphase is introduced ex situ through phenolic‐resin infiltration and controlled pyrolysis, establishing an interface‐design strategy that stabilizes fiber–matrix interactions while enabling efficient load transfer and thermal transport. The resulting C_f_/SiC exhibits benchmark mechanical robustness for this materials class. Physical vapor deposition (PVD) of dense Si and Ag films yields an ultrasmooth surface (0.031 *λ* in roughness) with high visible‐band reflectivity. By integrating additive shaping with interphase‐assisted densification and thin‐film finishing, this route enables systematic optimization of geometric formability and optical performance, with roughness and visible‐band reflectance set by the optical film stack and structural support from the C_f_/SiC substrate. It establishes a scalable paradigm for C_f_/SiC space mirrors while positioning interface‐engineered fabrication as a pathway for next‐generation multifunctional ceramic composites in aerospace environments.

## Graphical abstract

An integrated additive manufacturing route combines selective laser sintering, engineered interphases, and optimized silicon infiltration to fabricate complex C_f_/SiC composites. The approach enables precise shaping, high mechanical performance, and excellent optical reflectivity, offering a scalable solution for advanced aerospace optics with both structural robustness and surface smoothness.

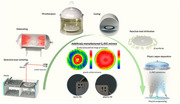

## 1 Introduction

The synergy of exceptional attributes exhibited by silicon carbide‐based composites endows them with distinctive properties, including thermal stability at elevated temperatures and ideal thermal conductivity (85–270 W mK^−1^).^[^ ^1^, ^2^ ^]^ In the cutting‐edge fields of heat exchange systems, nuclear energy systems, and lightweight space remote sensing optical components, carbon fiber reinforced silicon carbide (C_f_/SiC) composites, with their enhanced strength and improved toughness, are emerging as cornerstone materials that drive innovation and boost performance.^[^ ^3^, ^4^, ^5^, ^6^ ^]^ Nevertheless, the fabrication of large‐scale, lightweight, and complex‐shaped C_f_/SiC composites via conventional methods such as dry pressing, slip casting, and gel casting faces significant hurdles.^[^ ^7^, ^8^, ^9^ ^]^ These limitations stem from the inherent constraints of mold design and manufacturing capabilities, which struggle to accommodate the precise requirements of such advanced C_f_/SiC components.^[^ ^10^, ^11^ ^]^

While additive manufacturing, namely 3D printing, is a novel technique by stacking materials layer by layer to form various‐shaped components under a computerized automatic control system, which has been investigated, including binder jet printing (BJP), direct ink writing (DIW), stereolithography (SLA), and selective laser sintering.^[^ ^12^, ^13^ ^]^ Among them, SLS gains intensive attention due to its advantages of high efficiency, self‐supporting feature, and the convenience of large‐scale manufacturing.^[^ ^14^, ^15^ ^]^ In contrast to the direct near‐net‐shape SLS method applied to metals and polymers, composites reinforced with chopped‐carbon‐fiber require an indirect SLS approach, facilitated by a low‐melting‐point binder. Conversion of the printed green bodies into C_f_/SiC composites requires essential sintering processes such as liquid phase sintering (LPS), precursor infiltration and pyrolysis (PIP), and LSI.^[^ ^16^ ^]^ Chen et al.^[^ ^17^ ^]^ fabricated the continuous C_f_ reinforced SiC‐based composites from ink mixtures of SiC, α‐Al_2_O_3_, and Y_2_O_3_ mixed ink by DIW coupled with electric field‐assisted LPS without external pressure under 1900 °C. The composites exhibited excellent mechanical properties, with a density and flexural strength were 2.85 g cm^−3^ and 232 MPa, respectively. Zhang et al.^[^ ^18^ ^]^ prepared the SiC_p_/SiC composite lattice‐core sandwich panels by SLS and polycarbosilane (PCS) infiltration and pyrolysis for 8–10 cycles at 1200 °C. The open porosity, density, and compressive strength were 5.51%, 2.67 g cm^−3^, and 45.66 MPa, respectively. Our previous study reported a novel and efficient process to fabricate SiC composites via SLS from the powder mixtures of C_f_ and phenolic resin (PR) combined with one‐step LSI, with a density and strength of 2.719 ± 0.008 g cm^−3^ and 266 ± 5 MPa, respectively.^[^ ^14^ ^]^ Among these methods mentioned above, LSI demonstrates unique advantages of rapid densification under low temperature. However, high residual silicon in as‐sintered composites resulting from relatively high open porosity of printed bodies is the main weakness.

Recent studies have emphasized reducing porosity and tailoring carbon content in printed C_f_/SiC preforms as effective strategies to improve densification and overall composite performance. Zhu et al.^[^ ^19^ ^]^ fabricated the C_f_/SiC composite by hybrid techniques of SLS, one‐cycle PIP, and LSI, starting from PR‐coated C_f_ (PR@C_f_) composite powder. The density, flexural strength, and fracture toughness were 2.83 ± 0.03 g cm^−3^, 249 ± 17.0 MPa, and 3.48 ± 0.24 MPa m^1/2^, respectively. Wu et al.^[^ ^20^ ^]^ investigated the effects of impregnated PR concentration and PIP cycles on the properties of the Si/SiC ceramic matrix composites by SLS and LSI. The result indicated that the Si/SiC composites exhibited good mechanical properties. The density and flexural strength were 2.84 g cm^−3^, 180 MPa, and 2.96 g cm^−3^, 265 MPa, respectively, after PIP treatment with 66.7% vol PR‐ethanol solution for up to two cycles. In our previous study, SiC particles with excellent flowability were introduced into powder mixtures of C_f_ and PR prior to applying SLS and direct LSI without PIP treatment for the fabrication of SiC composites. The sintered density, flexural strength, and fracture toughness were 2.749 ± 0.006 g cm^−3^, 266 ± 5 MPa, and 3.30 ± 0.06 MPa m^1/2^, respectively, which were comparable to those of SiC composites with PIP treatment.^[^ ^21^ ^]^ Yet, the high residual Si content (> 50 vol%) was detrimental: since its properties were distinctively different from SiC, typically upon polishing the specimen, surface smoothness was difficult to enhance due to the co‐presence of dual phases with differed stiffness. Besides, the improvement of thermal properties was also critical for achieving thermal stability.

Moreover, the surface roughness of SLS‐fabricated C_f_/SiC composites falls short of space application requirements, limiting their current use to ground‐based optics and necessitating functional film deposition for space viability. In contrast, Li et al.^[^ ^22^ ^]^ fabricated SiC mirrors using fused deposition modeling–reactive melt infiltration, incorporating a laser‐melted transition layer and a post‐polished Ag film to achieve > 97% reflectivity in the 500–800 nm range. However, the rapid thermal cycling during laser cladding induces residual stress, necessitating a subsequent annealing step. Notably, 3D‐printed low‐defect C_f_/SiC mirror properties remain unreported. Regarding deposition techniques, physical vapor deposition (PVD) converts solids to vapor under vacuum to form dense, homogeneous, low‐defect films, with thickness and properties tailorable through parameter control (e.g., pressure, temperature, power). Similarly, Yang et al.^[^ ^23^ ^]^ employed compressive/tensile multilayer silicon films on SiC, achieving 0.69 nm by atomic force microscopy roughness and strong adhesion. However, C_f_/SiC fabrication constraints, particularly sub‐theoretical density and nonuniform phase hardness, combined with compromised polished surface quality, necessitate fully dense coatings. The CTE mismatch between silver (18–20 × 10^−6^  K^−1^) and C_f_/SiC composites (2–4 × 10^−6^  K^−1^) poses a significant risk of interfacial stress and surface distortion. To mitigate this, a silicon interlayer (2.6–4.0 × 10^−6^  K^−1^) is introduced, offering better thermal compatibility with the substrate.

**Figure 1:**
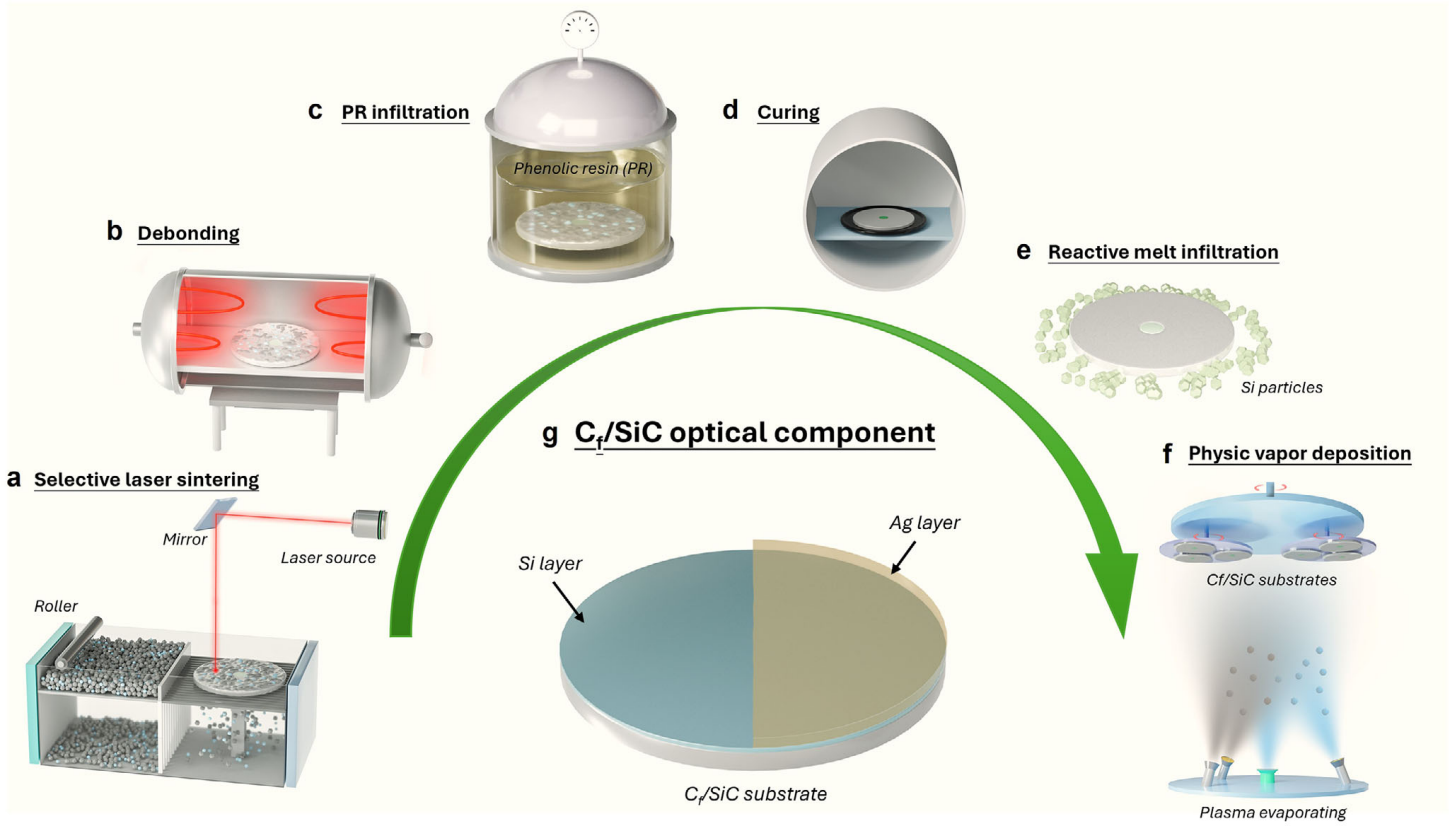
Schematic illustration of the integrated fabrication process for optical‐grade C_f_/SiC composites. The process includes selective laser sintering (SLS) of composites, phenolic resin (PR) infiltration and curing, debonding, reactive melt infiltration (RMI) with silicon particles, and surface modification via physical vapor deposition (PVD) to obtain the final optical component.

This study presents a unified manufacturing framework that bridges additive near‐net shaping with interfacial engineering and surface functionalization to fabricate aerospace‐grade C_f_/SiC optical components. By integrating SLS, precision‐controlled pre‐infiltration pyrolysis, and PVD‐based coating, the proposed route enables simultaneous optimization of structural integrity, thermal management, and optical reflectivity. The demonstrated synergy among architecture design, interface tailoring, and surface engineering not only enhances overall performance but also opens new possibilities for scalable, multifunctional ceramic systems. A schematic overview of the process is provided in **Figure** **1**, emphasizing the key stages of SLS‐based near‐net shaping using a powder roller and laser source, followed by post‐processing steps, and final surface modification through PVD via plasma evaporation under vacuum. These findings lay a foundation for the future development of lightweight, high‐precision optical structures in demanding environments such as space telescopes, high‐energy optics, and thermal protection systems. The Si/Ag coating stack sets microroughness and reflectance, while the C_f_/SiC substrate provides thermomechanical stability.

## 2 Results and Discussion

### 2.1 Microstructure and Mechanical Properties after PR Infiltration

**Figure 2:**
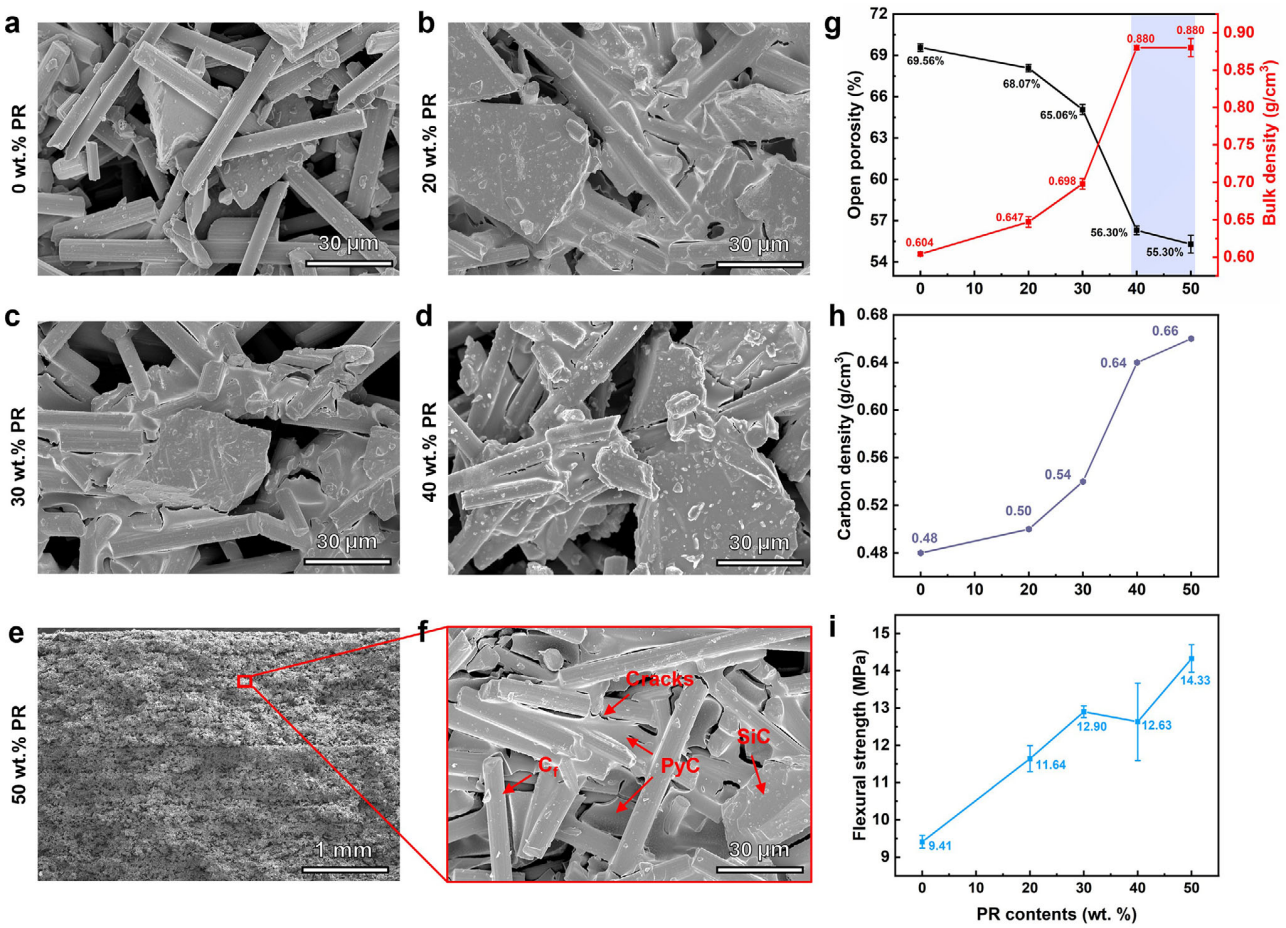
Correlation between microstructure and properties of PIP‐treated Cf/SiC/C composites with varying phenolic resin (PR) concentrations. a–f) SEM images of composites with 0, 20, 30, 40, and 50 wt.% PR, showing progressive improvements in matrix infiltration, structural continuity, and interfacial integrity. The detailed view in (f) reveals SiC formation, pyrolytic carbon (PyC) distribution, and residual cracks. g–i) Evolution of open porosity and bulk density (g), carbon density (h), and flexural strength (i) as a function of PR concentration. Increasing PR content markedly enhances densification and mechanical performance, with a significant transition observed above 30 wt.%.

The amount of PR added during precursor impregnation plays a crucial role in determining the distribution of PyC and the resulting microstructure of C_f_/SiC/C green bodies. As the PR addition increases, PyC deposition becomes more pronounced, effectively filling the interparticle pores between SiC particles and carbon fibers while simultaneously enhancing interfacial bonding. This leads to improved compactness and microstructural uniformity. With increasing PR addition from 0 wt.% to 50 wt.%, as shown in **Figure** **2**a–f, PyC gradually accumulates on the surfaces of SiC particles and carbon fibers, leading to a progressive rounding and roughening of particle edges. The originally sharp and well‐defined boundaries of C_f_ and SiC particles observed at 0 wt.% become increasingly coated and smoothed, indicating uniform PyC coverage and enhanced matrix continuity at higher addition levels. Although densification is significantly enhanced, microcracks are frequently observed after the PIP process. These cracks are primarily attributed to thermal expansion mismatch among carbon fibers, the SiC matrix, and the PyC interphase, particularly during cooling when differential contraction occurs. Notably, such microcracks may be beneficial for subsequent processing, as previous studies suggest they can serve as effective infiltration channels for molten silicon during LSI stage.

The evolution of porosity, bulk density, carbon density, and flexural strength of PIP‐treated C_f_/SiC/C samples as a function of PR addition is summarized in Figure 2g‐i. As shown in Figure 2g, increasing the PR content led to a marked reduction in open porosity and a corresponding increase in bulk density, primarily due to the pore‐filling effect of the resin‐derived pyrolytic carbon. These trends became less pronounced beyond 40 wt.% PR addition, indicating a near‐saturation state of available pore volume. A minimum open porosity of 55.30% was achieved at 50 wt.% PR addition. The observed porosity and density behavior is consistent with the microstructural evolution discussed previously.

The carbon density is an important factor for the subsequent LSI process, as shown in the following:^[^ ^24^ ^]^

(1) $$\rho_{c}=\left(\chi_{SiC}\frac{M_{c}}{M_{SiC}}+\chi_{C_{f}}+\chi_{PyC}\right)*\rho_{C_{f}/SiC/C}$$

where *ρ*_c_ and *ρ*_Cf/SiC/C_ are the carbon density and the density of PIP‐ed C_f_/SiC/C samples, respectively. *χ*_SiC_, *χ*_Cf_, *χ*_PyC_ are the mass percentage of the SiC, C_f_, and PyC, respectively. Carbon density *ρ*_c_ reflects both the amount of reactive carbon and the connectivity of the pore network formed after pyrolysis. When ρ_*c*_ is set just below ρ critical, a percolating network is preserved and carbon is sufficient to react; lower ρ_*c*_ risks carbon deficit, and higher ρ_*c*_ can constrict pore throats and cause pore blockage. *M*_C_, and *M*_SiC_ are the molecular mass of SiC and C, respectively. The calculated carbon density (*ρ*_c_) values are plotted in Figure 2h. With increasing PR addition, the PyC content increased correspondingly, leading to a steady rise in carbon density. A critical carbon density (*ρ*_critical_) of 0.963 g cm^−^^3^ has been reported, above which an entrapped carbon structure tends to form due to incomplete reaction between liquid silicon and carbon. Conversely, when *ρ*_c_ is below *ρ*_critical_, carbon can fully react with molten silicon, resulting in residual silicon within the final composite.^[^ ^25^ ^]^ In practice, volume expansion during the Si–C reaction may obstruct infiltration channels, which compromises the uniformity of the LSI process. To mitigate this, *ρ*_c_ should be carefully controlled slightly below *ρ*_critical_, thereby minimizing the risk of channel blockage and enabling more complete and homogeneous silicon infiltration. This principle was effectively implemented in the present study.

Improved flexural strength enhances structural integrity during LSI, enabling crack‐free fabrication of complex‐shaped components. As shown in Figure 2i, the flexural strength of PIP‐treated C_f_/SiC/C samples increased significantly with rising PR addition. This enhancement is primarily attributed to the reduced open porosity resulting from higher PyC content, which improves load transfer efficiency within the green body. At 50 wt.% PR, the flexural strength increased by 52% compared with the untreated sample, reaching a maximum of 14.33 ± 0.37 MPa.

### 2.2 Microstructure and Phase Composition of C_f_/SiC Composites

**Figure 3:**
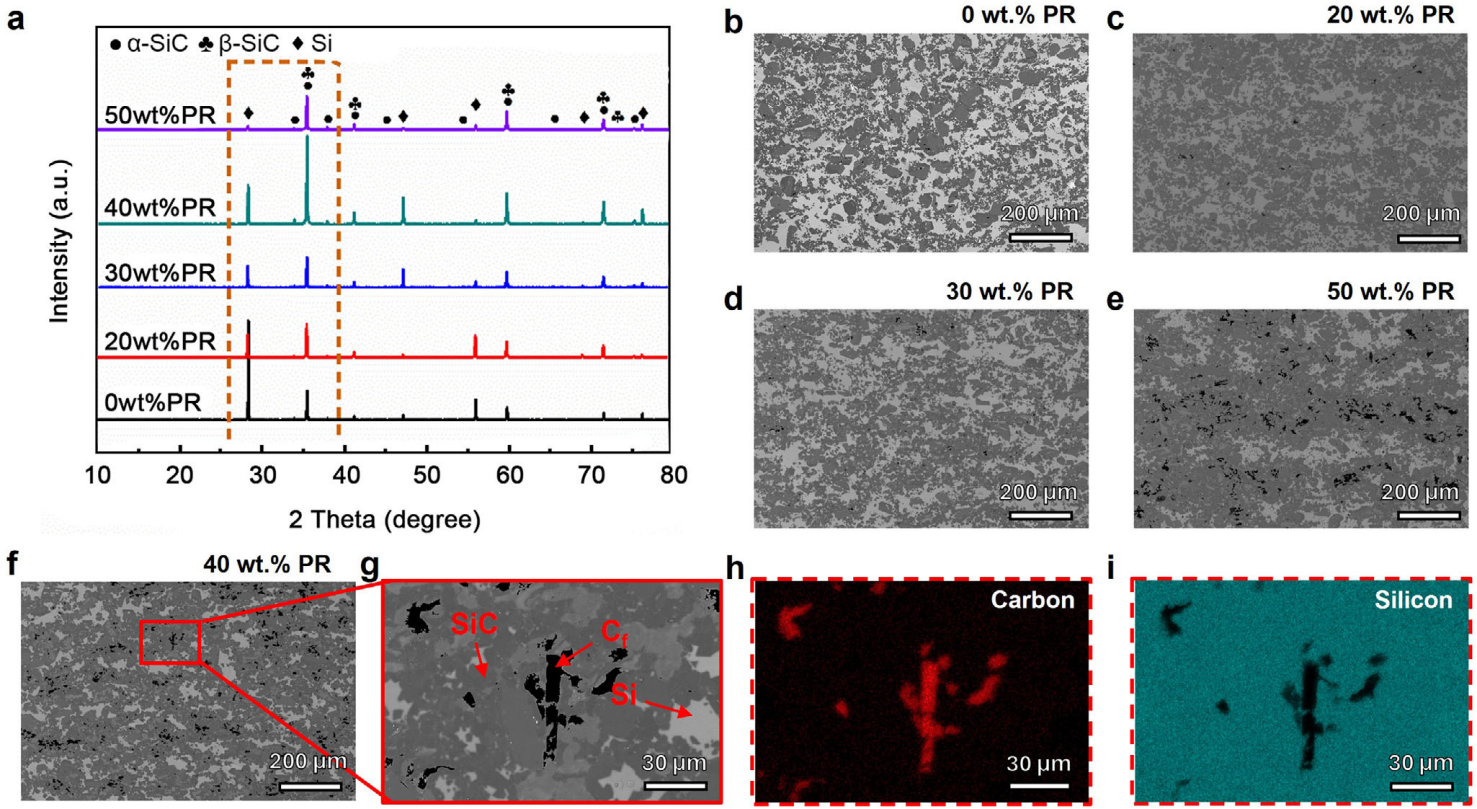
Phase composition and cross‐sectional microstructures of Cf/SiC/C composites treated with varying concentrations of phenolic resin. a) X‐ray diffraction (XRD) patterns showing the evolution of α‐SiC, β‐SiC, and residual Si phases with increasing PR addition. b–f) Back‐scattered electron (BSE) SEM images of polished cross‐sections reveal the distribution of SiC (dark gray), residual Si (light gray), and carbon (black). The carbon contrast includes C_f_, PyC derived from phenolic resin during the PIP process, and locally retained free carbon that remains after LSI. g–i) Enlarged BSE image and corresponding EDS maps of C and Si identifying the distribution of carbon‐rich and silicon‐rich regions.

The phase composition of C_f_/SiC composites fabricated with varying PR addition was analyzed by XRD, as shown in **Figure** **3**a. Three carbon forms are identified to maintain a consistent interpretation of the carbon‐related phases. C_f_ is introduced as the reinforcement during the SLS process and serves as the structural framework of the composite. PyC is generated from the decomposition of PR during the PIP stage. At lower temperatures, the PR‐derived carbon is amorphous, and it gradually transforms into turbostratic or partially graphitized carbon upon carbonization at 1100 °C under vacuum. Free carbon refers to the residual non‐fiber carbon that remains after the LSI process. During LSI at 1550 °C, PyC partially reacts with molten silicon to form a thin β‐SiC reaction interphase surrounding the fibers, while any unreacted PyC may persist locally as free carbon if infiltration is incomplete. Because some residual silicon is retained in the microstructure, most of the PyC is consumed during infiltration, whereas the carbon fibers remain structurally stable with a reacted interphase on their surfaces. The composites primarily consisted of pre‐added α‐SiC (6H), in situ formed β‐SiC (3C) generated from the reaction between carbon and molten silicon, and residual elemental silicon. The characteristic diffraction peaks of SiC and Si appeared at ≈2θ  =  35.70° and 28.55°, respectively. With increasing PR content, the intensity ratio of I_Si (28.55°)_ to I_SiC (35.70°)_ progressively declined, indicating a reduction in residual silicon and a corresponding increase in SiC formation. This trend confirms that higher carbon content facilitates a more complete reaction with molten silicon during LSI. Notably, no distinct carbon peaks were detected, likely due to overlap with adjacent SiC or Si signals.

The microstructure and elemental distribution of C_f_/SiC composites with varying PR additions are shown in Figure 3b‐i. The composites are primarily composed of SiC (dark gray), residual silicon (light gray), and carbon (black). As PR content increases from 0 to 50 wt.% (Figure 3b–f), the SiC phase becomes progressively more continuous and uniformly distributed, forming an interconnected network, while the residual silicon phase becomes increasingly discrete and sparse. This morphological evolution is consistent with the XRD results in Figure 3a, where a decreasing I_Si (28.55°)_ to I_SiC (35.70°)_ intensity ratio indicates enhanced conversion of carbon to SiC and reduced silicon retention. Notably, at higher PR concentrations, the carbon content on the polished surface also increases, as evidenced by the more prominent black regions. Figure 3g presents a high‐magnification image of the 40 wt.% sample, in which rod‐like carbon features ≈7 µm in diameter are observed, likely corresponding to exposed carbon fibers embedded in the SiC matrix. Elemental maps of carbon and silicon were collected by SEM‐EDS at 15 kV accelerating voltage with a probe current of ≈1 nA, a dwell time of 50 µs per pixel, and frame averaging to improve signal‐to‐noise. Elemental mapping (Figure 3g–i) confirms the spatial distribution of carbon and silicon, further verifying the compositional heterogeneity. These results highlight the role of PR addition in modulating the microstructure and phase distribution, ultimately improving the continuity and integrity of the SiC framework.

### 2.3 Mechanical and Thermal Properties of C_f_/SiC Composites

**Figure 4:**
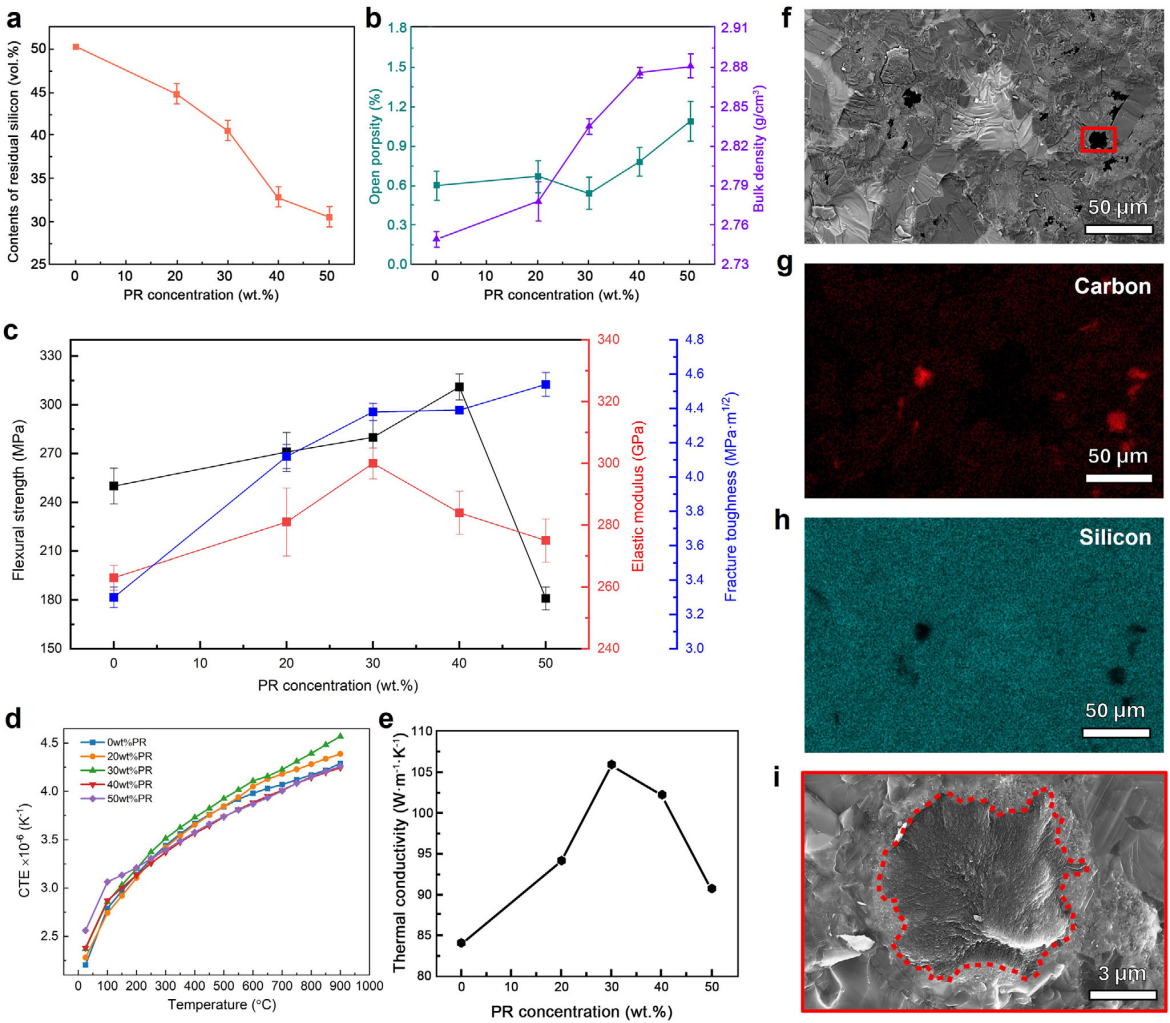
Mechanical, thermal, and fracture characteristics of C_f_/SiC composites with varying phenolic resin concentrations. a–e) Mechanical and thermal properties, including residual‐Si content, porosity, density, flexural strength, fracture toughness, elastic modulus, coefficient of thermal expansion (CTE), and thermal conductivity. f–i) Fractographic observations of the 40 wt.% PR sample showing the SiC matrix and embedded carbon fibers. The dark interfacial regions correspond to reacted or carbon‐rich interphases rather than fiber loss, consistent with controlled interfacial reactions that improve bonding.

The influence of PR addition on the mechanical and thermal properties of the C_f_/SiC composites is summarized in **Figure** **4**. As shown in Figure 4a, the residual silicon content decreased from 50.31 to 30.44 vol% as the PR concentration increased from 0 to 50 wt.%, with a marked plateau reached ≈40 wt.%. This trend is consistent with the XRD and microstructural observations (Figure 3a), confirming that increased PyC content facilitates more complete Si–C reactions during LSI. The reduction in residual silicon is primarily attributed to increased carbon availability and decreased porosity induced by PyC accumulation in the green bodies. Figure 4b further illustrates the porosity and bulk density variations. The open porosity remained below 1% for most samples, indicating nearly full densification, except for the 50 wt.% PR group (1.09 vol%), where excessive PyC likely caused closed pores and blocked infiltration pathways. As a result, bulk density increased with PR content, reaching a maximum of 2.881 ± 0.009 g cm^−^^3^ at 50 wt.%, in agreement with the increased SiC fraction and reduced free silicon.

The mechanical properties exhibited a strong correlation with the evolving microstructure. As shown in Figure 4c, flexural strength increased with PR addition and peaked at 311 ± 8 MPa at 40 wt.%, representing a 24.4% improvement over the non‐PIP sample (250 ± 11 MPa). This enhancement is attributed to improved interfacial bonding, increased SiC formation, and reduced residual Si content. However, at 50 wt.%, strength declined sharply to 181 ± 7 MPa due to microstructural inhomogeneity, closed pores, and isolated Si‐rich regions formed during LSI. Unlike strength, the fracture toughness exhibited a monotonic increase with PR content, reaching 4.54 ± 0.07 MPa·m^1/2^ at 50 wt.% PR, a 37.6% improvement. This may result from enhanced energy dissipation via fiber pull‐out and crack deflection, promoted by higher carbon content and PyC‐rich interfaces.^[^ ^26^ ^]^ The elastic modulus initially increased with PR addition, peaking at 300 ± 5 GPa at 30 wt.%, followed by a decline. As described by the empirical correlation below:^[^ ^27^ ^]^

(2) $$E=E_{0}\left(1-1.9P+0.9P^{2}\right)$$

where *E_0_* and *P* represent the elastic modulus of composites without porosity and porosity, respectively. That is, the elastic modulus increases with the decrease of porosity. Therefore, the elastic modulus in this study shows an opposite trend of the open porosity.

To further elucidate the origin of the enhanced fracture behavior, fracture surface analysis was conducted on the composite with 40 wt.% PR (Figure 4f‐i). The SiC matrix exhibited a transgranular fracture pattern, indicative of high matrix cohesion, while embedded chopped carbon fibers (≈5–6 µm in diameter) were partially wrapped by nanocrystalline β‐SiC (20–50 nm). This interfacial reaction zone, formed via controlled Si–C interaction during LSI, serves as a gradient transition layer that mitigates interfacial stress concentrations and suppresses decohesion. The nanocrystalline SiC layer likely results from a two‐stage reaction involving carbon dissolution into molten silicon, followed by β‐SiC nucleation and solid‐state interdiffusion. This tailored interphase enhances mechanical interlocking and crack deflection efficiency. Under external loading, cracks are repeatedly arrested or deflected at the fiber–matrix interface, enabling fracture energy dissipation via fiber pull‐out and interfacial debonding mechanisms well described by toughening models in ceramic matrix composites.^[^ ^28^, ^29^ ^]^ Moreover, the reduced fiber diameter relative to the nominal 7 µm provides direct evidence of silicon‐induced corrosion, which, while partially consuming the fiber surface, facilitates the formation of a tightly bonded reaction layer. These findings confirm that the PyC‐assisted interfacial architecture not only reinforces bonding but also introduces extrinsic toughening mechanisms, representing a strategic interface design pathway for enhancing the damage tolerance of C_f_/SiC composite in extreme environments.

Promising C_f_/SiC composites must exhibit not only high mechanical robustness but also excellent thermal dimensional stability to minimize distortion under fluctuating temperatures. The CTE of C_f_/SiC composites as a function of temperature and PR content is shown in Figure 4d. Across 100–200 °C, CTE values remain largely convergent, except for the 50 wt.% PR sample, which exhibits a higher value (3.2 × 10^−6^ K^−1^) due to increased free carbon content. In the elevated range of 200–900 °C, CTE increases nonlinearly, reaching 4.6 × 10^−6^ K^−1^ for the 30 wt.% PR composites.^[^ ^30^ ^]^ This variation reflects a compositional balance where the higher expansivity of SiC promotes thermal dilation, while the relatively low thermal expansion of carbon fibers and PyC mitigates it. These observations are consistent with earlier structural characterizations that confirmed the evolution of SiC and carbon content with PR concentration.

Thermal conductivity data are presented in Figure 4e. As the PR content increased from 0 to 30 wt.%, thermal conductivity improved markedly from 84.07 to 105.94 W·m^−1^·K^−1^, attributed to increased SiC formation and enhanced microstructural continuity. However, further increasing the PR content to 50 wt.% led to a decrease in conductivity to 90.74 W·m^−1^·K^−1^. This non‐monotonic behavior can be interpreted through Maxwell's effective medium theory, which relates thermal conductivity to the volume fractions and intrinsic conductivities of matrix and dispersed phases.^[^ ^31^ ^]^

(3) $$\lambda =\lambda_{m}\frac{2f_{p}\left(\lambda_{p}/\lambda_{m}-1\right)+\lambda_{p}/\lambda_{m}+2}{f_{p}\left(1-\lambda_{p}/\lambda_{m}\right)+\lambda_{p}/\lambda_{m}+2}$$

where *λ*_p_ and *λ*_m_ represent the thermal conductivity of the dispersed particles and matrix, respectively, *f*_p_ and *f*_m_ (*f*_m_ = 1‐*f*_p_) represent the volume fraction of the dispersed particles and matrix, respectively. Although SiC is a highly conductive phase, the concurrent increase in pyrolytic carbon (λ ≈ 3 W·m^−1^·K^−1^) and the emergence of closed porosity introduce phonon scattering centers that hinder thermal transport. Since thermal conduction in ceramic matrix composites is dominated by lattice vibration (phonons), the detrimental effects of PyC and pores outweigh the benefits of higher SiC content at excessive PR concentrations. This observation reinforces the critical balance between densification and microstructural uniformity in optimizing thermal transport properties.^[^ ^32^ ^]^

For mirror substrates, density sets the mass budget and gravitational sag. Flexural strength and fracture toughness define handling and vibration tolerance at the component scale. Thermal conductivity sets the thermal equalization time. CTE controls thermo‐elastic figure stability during thermal excursions. Root‐Mean‐Square (RMS) microroughness drives optical scattering and therefore visible‐band reflectance. Detailed numbers are provided in Table S1 (Supporting Information). Optical metrics are listed in Table S2 (Supporting Information).

### 2.4 Optical Properties of C_f_/SiC Component

**Figure 5:**
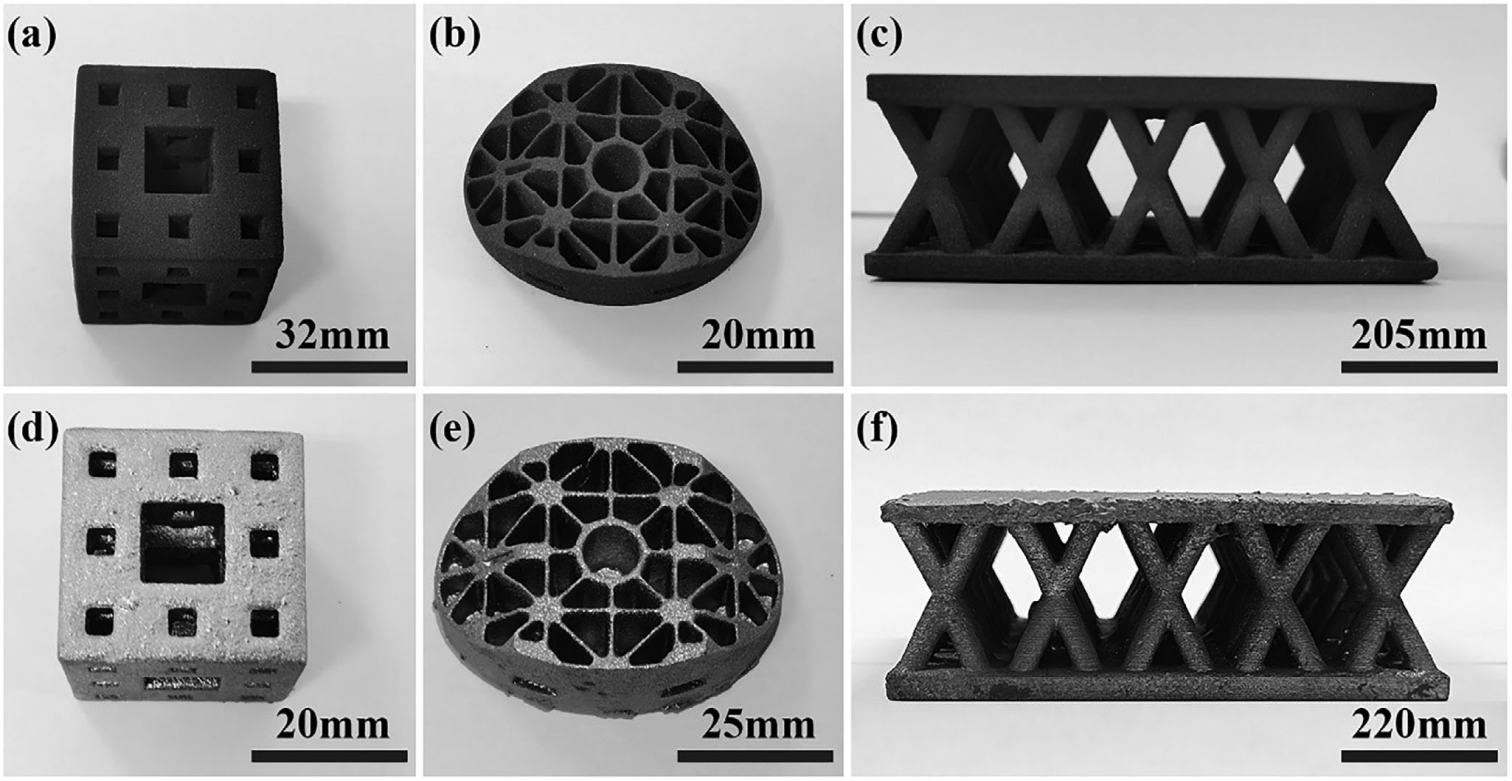
Selective laser sintering (SLS) of PIP‐derived Cf/SiC/C preforms and the resulting Cf/SiC composites with complex geometries (40 wt.% PR). a–c) Sintered PIP‐ed green bodies: (a) hollow cube, (b) optical component blank, and (c) lattice core sandwich panel. d–f) Corresponding Cf/SiC composites after reactive melt infiltration, retaining the designed structures with improved integrity and densification.

The geometric adaptability and structural integrity of the fabricated C_f_/SiC composites are demonstrated in **Figure** **5**, where complex structures including a hollowed cube, optical blank, and lattice‐core panel, were prepared from green bodies with 40 wt.% PR, retained their designed shapes after pyrolysis and infiltration without visible warping or cracking. This confirms the reliability of the SLS–PIP–LSI process for near‐net shaping of lightweight, intricate components.^[^ ^33^ ^]^ As summarized in Table S1 (Supporting Information), the present approach yields superior mechanical and thermal properties compared to other 3D‐printed SiC systems. Methods based on SLA and PCS often require multiple PIP cycles and still result in high porosity (7–15%), whereas our route achieves higher densification and better property integration with fewer steps.^[^ ^32^, ^34^, ^35^, ^36^, ^37^ ^]^ These results underscore the efficiency and scalability of this hybrid strategy for producing structurally and optically functional C_f_/SiC components.

**Figure 6:**
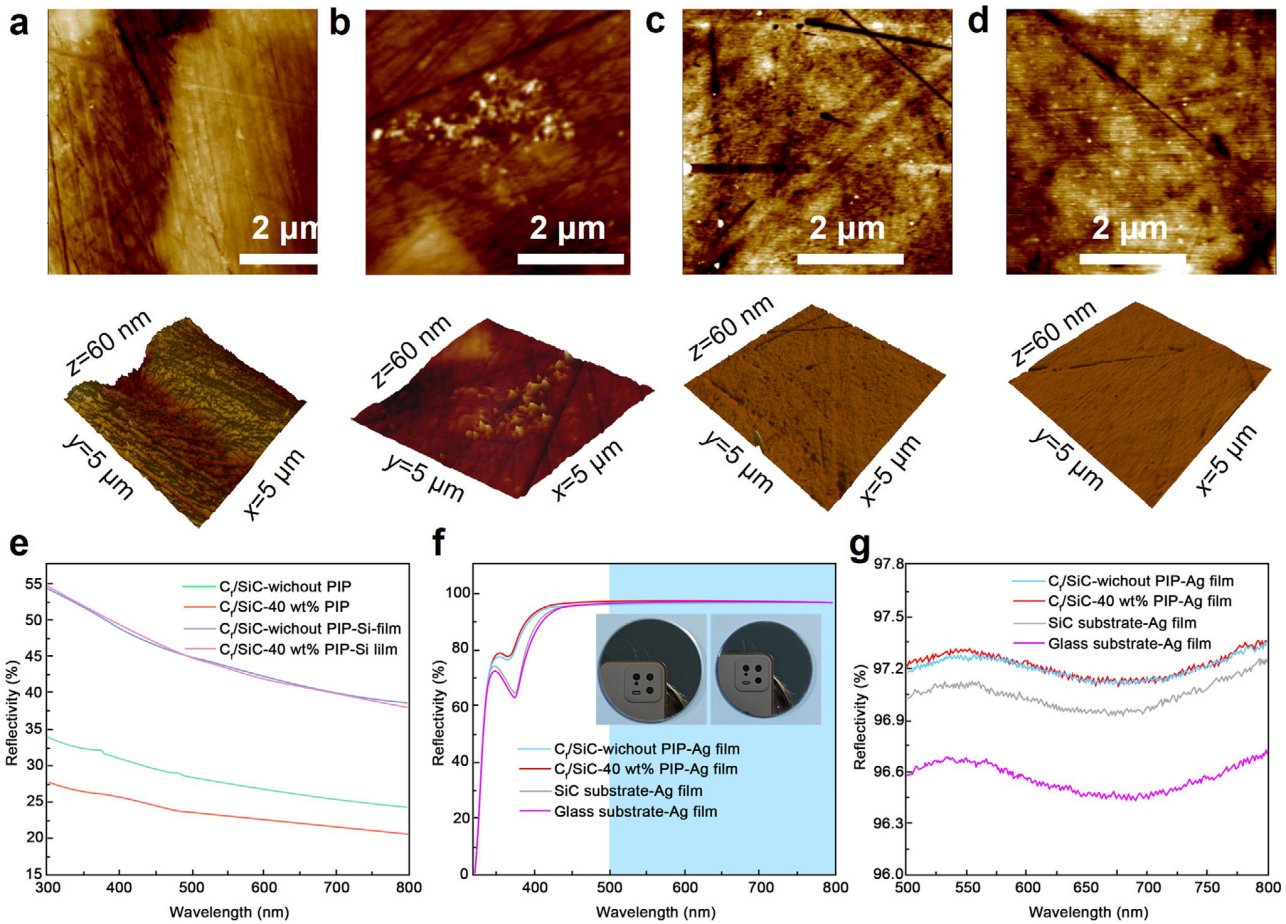
Surface morphology and optical reflectivity of Cf/SiC mirrors with and without PIP treatment. a–d) AFM images of Cf/SiC surfaces before (a,b) and after Si coating (c,d), without (a,c) and with (b,d) 40 wt.% PR PIP treatment. e) Reflectivity of substrates before and after Si deposition. f,g) Reflectivity after Ag coating across the full (f) and visible (g) spectral ranges. PIP treatment significantly improves surface uniformity and optical performance.

To address the limitations of direct polishing imposed by the heterogeneous hardness of carbon fiber, SiC, and residual silicon phases, a bilayer coating strategy was employed to achieve optical‐grade surfaces. A silicon transition layer was first deposited on the C_f_/SiC substrate using PVD, leveraging silicon's favorable wettability, thermal compatibility, and deposition accessibility. This Si film homogenizes the surface, enhancing subsequent machinability. As shown in **Figure** **6**a–d, the untreated C_f_/SiC substrate exhibits surface depressions due to differential abrasion during polishing. Atomic force microscopy (AFM) revealed that the average surface roughness (Ra) decreased from 4.62 nm (without PIP) to 3.99 nm (with 40 wt.% PR PIP), attributed to higher carbon density and reduced porosity, which promote SiC formation and limit residual silicon. This internal state carries to the surface after the Si transition layer: lower retained silicon yields a more uniform near‐surface, reduces hardness contrast and pull‐out, and lowers the post‐Si Ra to the sub‐nanometer range. After PVD Si deposition, the surface roughness was further reduced to 0.44 and 0.32 nm, respectively, for the untreated and PR‐treated samples, meeting the sub‐nanometer smoothness threshold required for spaceborne optical mirrors. Quantitative peak‐to‐valley (PV) surface figure, RMS, and Ra metrics are summarized in Table S2 (Supporting Information). In this wavelength range, surface roughness directly limits the absolute reflectance through scattering losses, whereas figure error chiefly degrades wavefront quality and energy concentration rather than the reflectance value itself.

For qualitative context, standard optical constants for Ag and Si in the visible band are used as a reference frame rather than as film‐specific fits.^[^ ^38^, ^39^ ^]^ The spectral and angular frame is near‐normal incidence over 300–800 nm, with band averages reported for 500–800 nm. Using the measured surface roughness of 0.031 *λ* (*λ* = 633 nm, red visible light), standard estimates indicate that roughness‐induced scattering is below 0.01% across this window, so roughness does not limit the mirrors reported here. The band‐averaged reflectance is ≈97.2% from 500 to 800 nm, and the remaining shortfall from the ideal thick‐Ag reference is attributed to intrinsic absorption in Ag together with the interface and morphology of the real films. Ag films can also show environment‐dependent Drude parameters near the visible, so tabulated constants are treated as a reference frame rather than a calibration for the present coatings.^[^ ^40^ ^]^

Silver is selected for the visible band on a physics basis. Its real permittivity is negative in 500–800 nm, and interband absorption is weak, because the main d‐to‐sp interband threshold lies in the near‐UV rather than in the visible.^[^ ^41^ ^]^ The extinction coefficient in this range is small. Once the Ag film is thicker than the visible skin depth, the reflectance approaches its intrinsic limit. Gold shows stronger interband loss toward the green and blue, which lowers its reflectance on the short‐wavelength side under the same thickness.^[^ ^42^ ^]^ Aluminum performs well into the near‐UV but has higher loss in the red visible and forms a stable native oxide.^[^ ^43^ ^]^ A Si transition layer improves wetting and adhesion on SiC, bridges CTE to Ag, and reduces interdiffusion at the interface. The basic loss account is as follows. Intrinsic absorption in Ag sets the ceiling. Residual scattering from microroughness reduces the measured reflectance. The Si layer lowers this scattering by homogenizing the near‐surface.

For visible‐band mirrors, silver was selected for its high reflectance among common metals, and a silicon transition layer was introduced to engineer the SiC interface. Silicon wets and adheres to SiC, bridges the thermal‐expansion difference to Ag, and limits interdiffusion; prior reports on Si interlayers and Ag mirrors on SiC support this stack.^[^ ^22^, ^23^ ^]^

Subsequently, a highly reflective Ag layer was deposited by PVD to form the final mirror surface. Figure 6e‐g compares the spectral reflectance across the 300–800 nm range. Across 500–800 nm, the band‐averaged reflectance showed no consistent trend with sample shape within the measurement uncertainty. Uncoated C_f_/SiC substrates exhibited modest reflectance (24.2%–38.8% for untreated, 20.7%–27.7% for PR‐treated), with the lower values in PR‐treated samples stemming from their higher carbon content, which increases surface absorption. In contrast, Ag‐coated mirrors achieved significantly improved reflectance exceeding 97% on average in the 500–800 nm band, regardless of PIP treatment. Notably, the PR‐treated sample exhibited slightly higher overall reflectivity, likely due to its improved interfacial continuity and surface morphology. These results demonstrate the viability of integrating SLS, interface tailoring, and PVD coatings to fabricate C_f_/SiC optical mirrors that meet stringent aerospace specifications. The inserted images in Figure 6f present photographs of the resulting optical components, which show uniform metallic luster and high reflectance.

**Figure 7:**
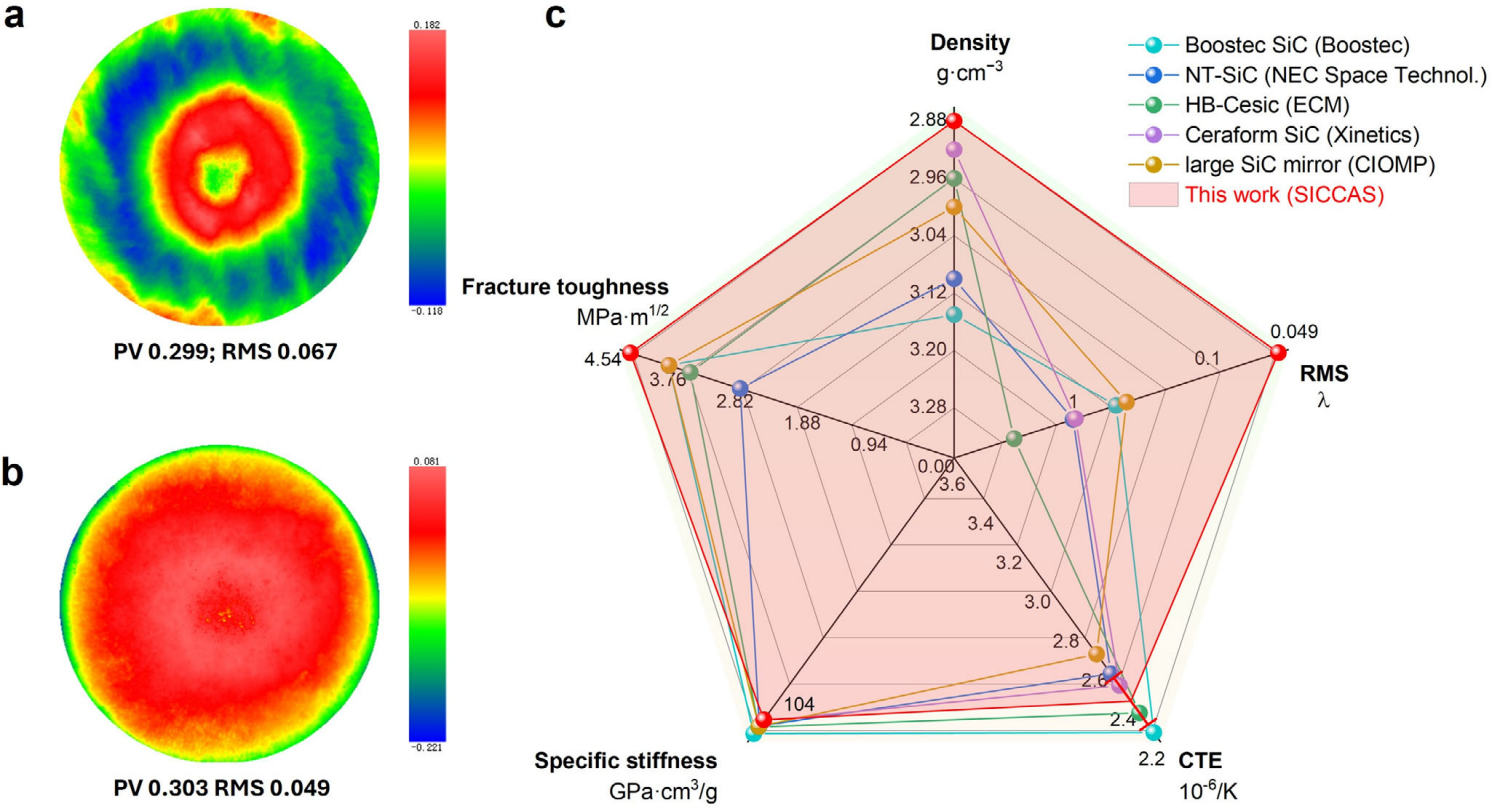
Surface wavefront maps (before PVD) and property benchmark of additively manufactured Cf/SiC mirrors. Full‐aperture interferometric figure maps for specimens without a) PR and with b) 40 wt.% PR, measured PV and map RMS are indicated on the maps. c) Radar comparison against representative SiC‐based mirrors (Boostec® SiC, NT‐SiC, HB‐Cesic®, Ceraform SiC, and CIOMP large SiC mirror).

**Figure** **7** reports the full‐aperture interferometric test results of the substrates after optical finishing (before PVD coating). For the 0 wt.% PR specimen, the surface figure is PV 0.299 *λ* and RMS 0.067 *λ* (Figure 7a), while for the 40 wt.% PR specimen, it is PV 0.303 *λ* and RMS 0.049 *λ* under identical conditions (Figure 7b). Although these metrics are nearly identical, the surface error map for the 40 wt.% PR specimen is visibly more uniform, particularly toward the periphery, indicating improved figure consistency without refiguring the low‐spatial‐frequency form of the substrate. By contrast, after coating deposition, the surface microroughness decreases to the sub‐nanometer regime, which sets the visible‐band scattering under near‐normal incidence. This superior uniformity is attributed to the higher elastic modulus, flexural strength, and fracture toughness of the 40 wt.% PR composite, which reduces local compliance and mitigates edge‐sensitive deformation during optical finishing, in this work (Figure 4c). The radar comparison in Figure 7c places the additively manufactured mirrors alongside representative SiC‐based mirrors and retains the mass and thermal advantages of the Cf/SiC bulk. A component‐level benchmark against representative SiC‐based mirrors from Boostec SiC, NT‐SiC, HB‐Cesic, Ceraform SiC, and CIOMP large SiC mirror is provided.^[^ ^44^, ^45^, ^46^, ^47^, ^48^ ^]^ Density influences areal mass efficiency and mitigates gravitational deformation, achieving a lightweight design with dimensional stability. Fracture toughness reflects the material's capability to resist crack formation and extension, ensuring structural reliability during manufacturing, assembly, and launch vibration conditions. RMS surface error represents the accuracy of the optical figure and has a direct impact on light scattering and imaging quality. The coefficient of thermal expansion guarantees thermoelastic consistency and maintains optical surface precision under temperature variations. Specific stiffness (*E/ρ*) describes the substrate's capacity to retain surface integrity under dynamic loads and to ensure stable structural resonance behavior. The additively manufactured mirrors in this research occupy the light end of density at ≈2.88 g·cm^−3^ and maintain good specific stiffness. Room temperature CTE locates in the range of 2.20 to 2.56 × 10^−6^  K^−1^ and fracture toughness is 4.54 MPa·m^1/2^. The surface RMS is 0.031 *λ*. This envelope aligns with or exceeds commercial ranges while preserving the geometric freedom enabled by additive manufacturing.

## 3 Conclusion

The fabrication route for C_f_/SiC optical components was systematically optimized by integrating selective laser sintering, polymer infiltration and pyrolysis, liquid silicon infiltration, and physical vapor deposition. The introduction of phenolic resin during the PIP stage enabled controlled pyrolytic carbon formation, which effectively reduced open porosity and enhanced the mechanical integrity of the green bodies. Post‐infiltration analysis revealed a significant decrease in residual silicon content, attributed to improved interfacial reactivity between molten silicon and the engineered carbon architecture. As a result, the composites exhibited a combination of high strength and fracture toughness for handling and vibration tolerance, while the deposition of dense Si and Ag films yielded ultrasmooth surfaces with an RMS value of 0.031 *λ* and visible‐range reflectivity averaging 97.2%. Taken together, our results deliver an innovation that achieves a practical separation of near‐net geometric formability from optical surface metrics using additive manufacturing. Microroughness and visible‐band reflectance are set by the Si/Ag coating stack, while thermomechanical stability is provided by the C_f_/SiC substrate. This innovative integration resolves the trade‐off among complex geometry, mechanical robustness, and optical finish in SiC composites and provides a practical path to lightweight, high‐precision mirrors for aerospace applications.

## 4 Experimental Section

### Raw Materials and Sample Preparation

The raw materials include commercially available SiC (D_50_ = 38.9 µm, Shanghai mechanical Co., Ltd.), chopped carbon fiber (200 mesh, Shanghai Composite Co., Ltd.), thermoplastic phenolic resin (PR) powders (D_50_ = 19.5 µm, Jinan Shengquan Material Factory, as binder) were applied for SLS. Thermoset liquid phenolic resin (Shanghai Adhesive Material Factory) was applied for the infiltration process, with ethanol (Analytical Reagent, Shanghai Lingfeng Chemical Reagent Co., Ltd.) as the solvent during infiltration. Additionally, commercial Si particles (2.33 g cm^−3^, average size: 1–3 mm) were used for LSI.

60vol% C_f_, 15vol% SiC, and 25vol% PR powders were ball‐milled at 66 rpm for 1 h using SiC media. SLS process was carried out using the selective laser printing equipment (Hunan Farsoon High‐Technology Co., Ltd., China). The laser power, scanning velocity, and hatch distance were 45 W, 7620 mm s^−1^, and 80 µm, respectively. The as‐printed bodies were heat‐treated at 1100 °C to remove organic binder and obtain porous C_f_/SiC/C green bodies under vacuum. Then, the C_f_/SiC/C green bodies were divided into four groups and were impregnated with the PR‐ethanol solution with varied impregnated PR concentrations of 20, 30, 40, and 50 wt.%, respectively. The impregnated samples were cured at 150 °C for 2 h, before being transferred and pyrolyzed at 1100 °C for 0.5 h under a vacuum atmosphere. Finally, C_f_/SiC composites were obtained via LSI at 1550 °C for 0.5 h under a vacuum atmosphere. During the PIP stage, the PR decomposes and carbonizes to form PyC that coats both SiC particles and carbon fibers. This PyC provides a carbon source for the subsequent Si–C reaction during the LSI process. At 1550 °C, part of the PyC reacts with molten silicon to generate a thin β‐SiC layer at the fiber–matrix interface, while unreacted carbon may remain as isolated free carbon in regions where infiltration is locally limited.

The C_f_/SiC composite substrate was ultrasonically cleaned for 30 min in ethanol before the deposition of Si and Ag layers by electron beam with the aid of ion beam assisted deposition (Ares1110, Leybold Optics, Germany) on K9 glass and monocrystalline silicon substrates at 100 °C. The bulk material was heated by an electron gun until vapor was deposited onto the substrate surface, during which the ion beam is continuously bombarded to obtain denser films.

### Characterization

A scanning electron microscopy (SEM, SU8220, Hitachi, Japan), along with energy dispersive spectrometry (EDS) were used to observe the morphology, microstructure elemental compositions. The bulk density and open porosity of C_f_/SiC/C green bodies and C_f_/SiC composites were determined based on Archimedes principle. The phase compositions of C_f_/SiC composites were analyzed by X‐ray diffractometer with Cu Kα radiation (XRD, 40 KV, 40 mA, D/max 2550 V, Japan). The volume fraction of the residual silicon was measured by HF‐HNO_3_ mixed acid etching method.

Carbon density ρ_c_ is the mass of carbon per unit preform volume before LSI, expressed in g·cm^−3^. It was obtained from the measured preform mass *m*_preform_(g), the mass fraction of the SiC skeleton ω_SiC_(dimensionless), the mass fraction of the mixed carbon sources introduced before LSI ω_mixedC_(dimensionless), and the preform volume *V*_preform_(cm^3^). SiC was converted to its carbon equivalent and added to the mixed carbon:^[^ ^24^, ^49^ ^]^

(4) $$\rho_{c}=\frac{m_{preform}\cdot \omega_{SiC}\cdot \left(M_{C}/M_{SiC}\right)+m_{preform}\cdot \omega_{mixedC}}{V_{preform}}$$

*M_C_* and*M*_SiC_are the molar masses of carbon and stoichiometric SiC (g·mol^−1^). ω_mixedC_ included PR uptake multiplied by its carbon yield at the pyrolysis temperature and any PyC addition. ρ_c_ was used as a process‐setting metric. Targeting ρ_c_ just below the empirically identified ρcritical maintained a percolating pore network while avoiding carbon deficit.

The flexural strength and elastic modulus were measured by a three‐point bending test (Instron‐1195, Instron, USA) with a sample size of 4 mm × 3 mm × 36 mm. The fracture toughness (*K*_IC_) was measured by the single‐edge notched beam (SENB) method with a sample size of 2.5 mm × 5 mm × 36 mm. The thermal diffusivity (*a*) was tested at 25 °C using a laser‐flash technique (TA DLF2800, America). The specific heat (*C_p_*) at 25 °C was measured using a differential scanning calorimeter (DSC; STA 449 F1 Jupiter Netzsch, Selb, Germany). The thermal conductivity (*k*) at 25 °C can be calculated using the following:^[^ ^50^ ^]^

(5) $$k=a\cdot \rho \cdot C_{p}$$

where *a* is the thermal diffusivity, *ρ* is the bulk density, and *C_p_* is the specific heat capacity.

AFM experiments were conducted using Vero ES AFM (Oxford Instrument, UK). Surface roughness was measured by interferometry in accordance with the standard GJB 9248‐2017. Three spots per sample were measured. Results were reported as mean ± one standard deviation, or to the instrument resolution if a single location was available. The film surface fluctuations and roughness (Ra) were measured by the zygo laser interferometers (Zygo; USA). The reflectivity was tested using the ultraviolet–visible (UV–vis) spectrophotometer (Lambda 950; PerkinElmer, USA). Reflectance measurements followed the standard ZWB 230‐2006. Reflectance spectra were recorded at an incidence angle of 8° over the 300–800 nm visible range. The band‐averaged reflectance was calculated as the arithmetic mean of R(λ) from 500 to 800 nm at the instrument's sampling interval. Numerical precision was rounded accordingly.

Full‐aperture figure maps were acquired in accordance with GB/T 2831‐2009 using a 4‐inch 633 nm Fizeau interferometer. The reported figure metrics were PV and map RMS from the full pupil. Microroughness was measured by interferometry in accordance with GJB 9248‐2017. Three locations per sample were measured for each modality.

## Conflict of Interest

The authors declare no conflict of interest.

## Supporting information



Supporting Information

## Data Availability

The data that support the findings of this study are available from the corresponding author upon reasonable request.
