# Supplementary material for: From Complex Shaping to Mirror Finish: Additive Manufacturing of Aerospace‐grade Cf/SiC Space Optics
Source: Adv Sci (Weinh). 2025 Nov 8;13(6):e17980. doi: 10.1002/advs.202517980 (PMC12866843; doi:10.1002/advs.202517980)
Supplement: Supplementary file 1 — Supporting Information [file ADVS-13-e17980-s001.docx]

From complex shaping to mirror finish: additive manufacturing of aerospace-grade C_f_/SiC space optics

*Buhao Zhang et al.*

*Corresponding author. Email: jieyin@mail.sic.ac.cn; xjliu@mail.sic.ac.cn; zhrhuang@mail.sic.ac.cn

**This PDF file includes:**

Tables S1 to S2

**Table S1. Properties comparison of the SiC composites fabricated by 3D printing and PIP.**

| Methods | Precursor/cycles | Materials | Density  (g⋅cm^−3^)/ Relative density (%) | Flexural strength  (MPa) | Fracture toughness  (MPa·m^1/2^) | Thermal conductivity  (W·m^−1^·K^−1^) | References |
| --- | --- | --- | --- | --- | --- | --- | --- |
| SLS+PIP+LSI | PR / 1 cycle | C_f_/SiC | 2.88 | 311 | 4.54 | 105.94 | This work |
| SLS+PIP+LSI | PR / 1 cycle | C_f_/SiC | 2.83 | 249 | 3.48 | - | [19] |
| SLS+PIP+LSI | PR / 1 cycle | C_f_/SiC | 2.89 | 237 | 3.56 | 84 | [32] |
| SLS+PIP+LSI | PR / 2 cycles | Si/SiC | 2.96 | 265 | - | - | [20] |
| SLS+PIP+LSI | PR / 2 cycle | Graphite/SiC | 2.46 | 202.10 | - | 104.21 | [30] |
| SLA+PIP | PCS / 8 cycles | SiC | 84.8% | 204 | - | - | [33] |
| SLA+PIP | PCS / 8 cycles | SiC | 93.5% | 165.2 | - | - | [34] |
| SLA+PIP+LSI | PR / 1 cycle | SiC | 2.89 | 244 | - | - | [22] |
| DIW+LSI | - | SiC | 2.94 | 224 | - | 112 | [35] |
| LPBF+PIP+LSI | PCS / 1 cycle | C_f_/SiC | 2.96 | 257.11 | 3.60 | - | [36] |
| FDM+PIP+LSI | PR / 4 cycles | SiC_f_/SiC | - | 398 | 10.79 | - | [37] |

**Table S2. PV, RMS, and Ra of the Cf/SiC optical component**

| Samples | Substrate | | | After depositing Si layer | | |
| --- | --- | --- | --- | --- | --- | --- |
|  | PV/λ | RMS/λ | Ra/nm | PV/λ | RMS/λ | Ra/nm |
| 0wt%PR | 0.299 | 0.067 | 4.62 | 0.174 | 0.021 | 0.437 |
| 40wt%PR | 0.303 | 0.049 | 3.99 | 0.316 | 0.031 | 0.323 |
